# Active and passive smoking impacts on asthma with quantitative and temporal relations: A Korean Community Health Survey

**DOI:** 10.1038/s41598-018-26895-3

**Published:** 2018-06-05

**Authors:** So Young Kim, Songyong Sim, Hyo Geun Choi

**Affiliations:** 1 Department of Otorhinolaryngology-Head & Neck Surgery, CHA Bundang Medical Center, CHA University, Seongnam, Korea; 2 Department of Statistics, Hallym University, Chuncheon, Korea; 3 Department of Otorhinolaryngology-Head & Neck Surgery, Hallym University Sacred Heart Hospital, Anyang, Korea

## Abstract

This study aimed to evaluate the relations of smoking with asthma and asthma-related symptoms, considering quantitative and temporal influences. The 820,710 Korean adults in the Korean Community Health Survey in 2009, 2010, 2011, and 2013 were included and classified as non-smoker, past smoker or current smoker. Total smoking years, total pack-years, and age at smoking onset were assessed. Information on wheezing, exercise wheezing, and aggravation of asthma in the past 12 months and asthma diagnosis history and current treatment was collected. Multiple logistic regression analysis with complex sampling was used. Current and former smokers showed significant positive relations with wheezing, exercise wheezing, asthma ever, current asthma, and asthma aggravation. Current smokers demonstrated higher adjusted odd ratios (AORs) for wheezing, exercise wheezing, and asthma aggravation than former smokers. Former smokers showed higher AORs than current smokers for current asthma treatment. Longer passive smoking was related to wheezing and exercise wheezing. Greater age at smoking onset and duration since cessation were negatively related to wheezing, exercise wheezing, and current asthma; total pack-years demonstrated proportional associations with these symptoms. Former, current, and passive smoking was positively correlated with wheezing and exercise wheezing. Total pack-years and early initiation were increasingly related to asthma.

## Introduction

Smoking is one of the preventable risk factors for asthma^1^. Many previous studies have evaluated the impacts of smoking on asthma^2,3^. Because the patterns of smoking are diverse, including current vs. previous and active vs. passive, the impact of smoking can vary. Active smoking was suggested to have adverse effects on asthma to various degrees ranging from its occurrence to aggravation^4,5^. Active smoking was associated with a 1.36-fold increase in the development of asthma in a prospective study^4^. Approximately 36% (confidence intervals [95% CI] = 34% − 38%) of emergency department patients with asthma aggravation were current smokers^6^. The adverse effects of smoking could be sustained after smoking cessation. Former smokers also reported a aggravation and persistent asthma^7,8^. Although there have been several reports on the prolonged risk of asthma in former smokers, few studies have investigated the differential adverse effects of smoking status on the symptoms or diagnosis of asthma^7–9^. Like active smoking, passive smoking has also been reported to increase the risk of the development or aggravation of asthma^4,10,11^. Even in subjects who have never experienced active smoking, subjects with passive smoking have been shown to have an elevated development of asthma, with a hazard ratio of 1.21 compared with never-active non-smokers^4^.

Smoking patterns (e.g., current or previous, active or passive) are reciprocally correlated. Active smokers harmfully affect their family and neighbors by provoking the initiation of smoking and by emitting second-hand tobacco smoke. It has been reported that parental smoking and friend smoking increased current smoking in adolescents up to 24.18 times^12^. Therefore, these entangled smoking statuses of current, former, active and passive smoking should be independently considered to elucidate the effects of each smoking status on asthma. Additionally, susceptibility to the adverse effects of smoking on asthma has been suggested to occur more frequently at an early age. Prospective studies demonstrated that perinatal smoking exposure resulted in greater development of asthma and decreased lung function than that resulting from later exposure to smoking^13^; however, only a few previous studies evaluated the impact of the quitting period or the age of exposure to smoking on asthma.

The purpose of the present study was to delineate the effects of various smoking patterns on asthma using large, nationwide, population-based data. We evaluated the quantitative effects of smoking with respect to the duration, amount, and age of onset of smoking. Furthermore, the impact of smoking on asthma was extensively investigated using several asthma-related parameters, including wheezing, exercise wheezing, and exacerbation of asthma. The potential confounders of age, region of residence, type of housing, income level, education level, physical activity, obesity, sleep time, stress level, and alcohol intake were adjusted for to evaluate the relationship between smoking and asthma in this study^1^. In addition, various smoking status and asthma-related parameters were also reciprocally adjusted for using multiple logistic regression analysis.

## Results

A total of 60.6% (510,750/820,710), 13.8% (119,062/820,710), and 25.6% (190,898/820,710) of the participants were non-smokers, former smokers, and current smokers, respectively (Table 1). Additionally, 11.1% and 21.9% of the participants were exposed to passive smoking at home and in the workplace, respectively. Approximately 2.4% and 2.5% of the participants experienced wheezing and exercise wheezing, respectively, in the past year, 2.1% of the participants had a history of asthma diagnosed by medical doctors, and 0.7% of the participants currently suffered from asthma. Among the participants who had current asthma, 22.7% experienced aggravation of asthma within the past year. The average age of all the participants was 44.9 years, and 50.2% of them were male. The number of occupants in the household, type of house, levels of income and education, physical activity, obesity, sleep time, level of stress, and alcohol consumption differed according to smoking status. Thus, all these variables were adjusted as covariates.

**Table 1:** Baseline characteristics of participants.

	Total	Non-smoker	Past smoker	Current smoker
Number (%^*^)	820,710 (100)	510,750 (60.6)	119,062 (13.8)	190,898 (25.6)
Age, year, mean (SD)	44.9 (16.4)	44.3 (16.9)	52.5 (14.8)	42.3 (14.7)
Sex, n (%^*^)				
Male	383,389 (50.2)	95,591 (22.2)	111,356 (93.1)	176,442 (93.3)
Female	437,321 (49.8)	415,159 (77.8)	7,706 (6.9)	14,456 (6.7)
Region, n (%^*^)				
Urban	252,848 (47.3)	159,596 (47.8)	35,016 (47.4)	58,236 (46.1)
Rural	567,862 (52.7)	351,154 (52.2)	84,046 (52.6)	132,662 (53.9)
Household, n, mean (SD)	3.3 (1.3)	3.3 (1.4)	3.2 (1.3)	3.2 (1.3)
House type, n (%^*^)				
A detached house	358,169 (26.7)	220,808 (26.3)	57,065 (28.3)	80,296 (26.9)
Condominium	308,876 (49.9)	197,496 (51.4)	42,665 (50.9)	68,715 (45.8)
Others	153,665 (23.4)	92,446 (22.3)	19,332 (20.8)	41,887 (27.3)
Income, n (%^*^)				
Lowest	174,688 (13.6)	110,405 (13.8)	29,067 (15.0)	35,216 (12.2)
Low-middle	222,917 (25.3)	137,594 (25.2)	31,531 (24.4)	53,792 (26.1)
Upper-middle	215,386 (29.4)	132,783 (29.1)	28,672 (27.8)	53,931 (31.1)
Highest	207,719 (31.7)	129,968 (31.9)	29,792 (32.7)	47,959 (30.6)
Education, n (%^*^)				
Low	297,969 (23.0)	200,060 (25.2)	47,296 (25.9)	50,613 (16.4)
Middle	249,711 (31.7)	140,946 (29.1)	37,046 (33.1)	71,719 (37.3)
High	273,030 (45.2)	169,744 (45.7)	34,720 (41.0)	68,566 (46.4)
Physical activity, n (%^*^)				
Low (0 min/week)	466338 (56.2)	306,734 (59.8)	60,543 (48.7)	99,061 (52.0)
Middle (1–149 min/week)	58328 (8.4)	37,773 (8.8)	7,821 (7.9)	12,734 (7.7)
High (≥150 min/week)	296044 (35.4)	166,243 (31.5)	50,698 (43.4)	79,103 (40.3)
Obesity, n (%^*^)				
Underweight (<18.5 kg/m^2^)	46,170 (5.7)	34,519 (7.3)	4,209 (2.6)	7,442 (3.3)
Healthy(≥18.5, <25.0 kg/m^2^)	582,531 (71.1)	370,387 (73.3)	79,677 (66.2)	132,467 (68.3)
Overweight (≥25, < 30.0 kg/m^2^)	175,244 (21.1)	96,161 (17.4)	32,884 (29.2)	46,199 (25.6)
Obese (≥30.0 kg/m^2^)	16,765 (2.1)	9,683 (1.9)	2,292 (2.1)	4,790 (2.8)
Sleep time, n (%^*^)				
≤5 hour/day	123,462 (14.7)	77,726 (14.7)	18,072 (14.7)	27,664 (14.6)
6 hour/day	232,958 (30.4)	141,564 (29.3)	34,335 (32.0)	57,059 (32.2)
7 hour/day	263,598 (32.7)	165,096 (32.9)	37,069 (32.3)	61,433 (32.3)
8 hour/day	165,589 (18.5)	104,722 (19.2)	23,948 (17.5)	36,919 (17.3)
≥9 hour/day	35,103 (3.8)	21,642 (3.9)	5,638 (3.5)	7,823 (3.6)
Stress level, n (%^*^)				
No	169,742 (16.9)	105,138 (17.4)	31,355 (21.0)	33,149 (13.6)
Some	436,459 (54.9)	279,057 (56.7)	61,329 (54.1)	96,073 (51.3)
Moderate	187,866 (24.6)	111,769 (22.9)	23,259 (21.9)	52,838 (30.0)
Severe	26,643 (3.5)	14,786 (3.0)	3,019 (2.9)	8,838 (5.1)
Alcohol, n (%^*^)				
≤1 time a month	391,757 (41.6)	305,765 (53.8)	42,307 (29.5)	43,685 (19.2)
2–4 times a month	253,714 (36.2)	156,174 (36.1)	34,604 (34.8)	62,936 (37.0)
≥2 times a week	175,239 (22.3)	48,811 (10.1)	42,151 (35.7)	84,277 (43.8)
Passive smoking at home, n (%^*^)				
0 hour/day	733,031 (88.9)	451,960 (88.1)	115,041 (96.3)	166,030 (86.8)
<1 hour/day	74,147 (9.5)	50,359 (10.3)	3,538 (3.3)	20,250 (10.8)
≥1 hour/day	13,532 (1.6)	8,431 (1.5)	483 (0.4)	4,618 (2.4)
Passive smoking at workplace, n (%^*^)				
0 hour/day	663,138 (78.1)	443,672 (85.6)	94,799 (74.6)	124,667 (62.2)
<1 hour/day	118,514 (16.3)	53,155 (11.5)	19,255 (19.9)	46,104 (25.9)
≥1 hour/day	39,058 (5.6)	13,923 (3.0)	5,008 (5.5)	20,127 (11.9)
Cigarette, mean (SD)			16.7 (11.2)	15.3 (8.8)
Total smoking time, year, mean (SD)			18.6 (13.7)	21.9 (14.0)
Total pack-year, mean (SD)			17.8 (20.8)	17.7 (17.7)
Period of quitting smoking, year, mean (SD)			13.3 (11.6)	
Starting age of smoking, year, mean (SD)			20.5 (5.1)	20.4 (5.7)
Wheezing within 1 year, n (%*)				
Yes	23,487 (2.4)	12,336 (2.0)	4,435 (2.8)	6,716 (3.0)
No	797,223 (97.6)	498,414 (98.0)	114,627 (97.2)	184,182 (97.0)
Exercise wheezing within 1 year, n (%*)				
Yes	24,291 (2.5)	13,162 (2.2)	4,660 (3.0)	6,469 (2.9)
No	796,419 (97.5)	497,588 (97.8)	114,402 (97.0)	184,429 (97.1)
Asthma ever, n (%^*^)				
Yes	20,331 (2.1)	11,820 (2.1)	4,302 (2.8)	4,209 (1.9)
No	800,379 (97.9)	498,930 (97.9)	114,760 (97.2)	186,689 (98.1)
Current asthma, n (%^*^)				
Yes	8,897 (0.7)	4,709 (0.7)	2,378 (1.3)	1,810 (0.6)
No	811,813 (99.3)	506,041 (99.3)	116,684 (98.7)	189,088 (99.4)
Asthma aggravation within 1 year, n (%^*^)				
Yes	2,161 (22.7)	1,094 (22.1)	580 (23.0)	487 (24.2)
No	6,736 (77.3)	3,615 (77.9)	1,798 (77.0)	1,323 (75.8)

^*^Estimated prevalence adjusted recommended weighted value.

Current smokers demonstrated higher AORs (95% confidence interval [CI]) for wheezing (2.07 [1.90 to 2·25]; 2.42 [2.20 to 2.67]) and exercise wheezing (1.68 [1.54 to 1.83]; 2.09 [1.90 to 2.31]) than non-smokers and former smokers among both workers and non-workers. Current smokers had a higher AOR for asthma aggravation (1.64 [1.27 to 2.12]) among workers. Former smokers had higher AORs for current asthma (1.74 [1.48 to 2.04]; 2.24 [1.93 to 2.60]) than non-smokers and current smokers among both workers and non-workers (Table 2).

**Table 2:** Adjusted odd ratios of smoking status and passive smoking for asthma related questions in worker and non-worker.

	Wheezing		Exercise wheezing		Asthma ever		Asthma current		Asthma aggravation	
	OR (95% CI)^†^	*P*-value	OR (95% CI)^†^	*P*-value	OR (95% CI)^†^	*P*-value	OR (95% CI)^†^	*P*-value	OR (95% CI)^†^	*P*-value
**Worker (n = 551**,**801)**										
Active smoking status		<0.001^*^		<0.001^*^		<0.001^*^		<0.001^*^		<0.001^*^
Non-smoker	1		1		1		1		1	
Past smoker	1.43 (1.30 to 1.58)		1.39 (1.26 to 1.53)		1.45 (1.32 to 1.60)		1.74 (1.48 to 2.04)		1.57 (1.20 to 2.05)	
Current smoker	2.07 (1.90 to 2.25)		1.68 (1.54 to 1.83)		1.17 (1.06 to 1.29)		1.33 (1.13 to 1.57)		1.64 (1.27 to 2.12)	
Passive smoking, home		<0.001^*^		<0.001^*^		<0.001^*^		<0.001^*^		0.864
0 hour/day	1		1		1		1		1	
<1 hour/day	1.22 (1.11 to 1.33)		1.31 (1.21 to 1.43)		1.12 (1.02 to 1.24)		1.22 (1.03 to 1.44)		1.02 (0.75 to 1.39)	
≥1 hour/day	1.63 (1.39 to 1.91)		1.65 (1.42 to 1.93)		1.39 (1.14 to 1.70)		1.62 (1.18 to 2.21)		0.92 (0.67 to 1.27)	
Passive smoking, work		<0.001^*^		<0.001^*^		0.043^*^		0.861		0.844
0 hour/day	1		1		1		1		1	
<1 hour/day	1.09 (1.02 to 1.16)		1.17 (1.09 to 1.24)		0.99 (0.92 to 1.06)		1.04 (0.91 to 1.18)		1.04 (0.83 to 1.31)	
≥1 hour/day	1.51 (1.39 to 1.65)		1.59 (1.46 to 1.73)		1.13 (1.02 to 1.26)		1.03 (0.84 to 1.25)		0.95 (0.71 to 1.26)	
**Non-worker (n = 268**,**909)**										
Smoking status		<0.001^*^		<0.001^*^		<0.001^*^		<0.001^*^		0.555
Non-smoker	1		1		1		1		1	
Past smoker	1.77 (1.59 to 1.95)		1.75 (1.58 to 1.93)		1.78 (1.60 to 1.98)		2.24 (1.93 to 2.60)		0.98 (0.81 to 1.19)	
Current smoker	2.42 (2.20 to 2.67)		2.09 (1.90 to 2.31)		1.67 (1.48 to 1.86)		2.07 (1.76 to 2.43)		1.10 (0.90 to 1.34)	
Passive smoking, home		<0.001^*^		<0.001^*^		0.042^*^		0.286		0.861
0 hour/day	1		1		1		1		1	
<1 hour/day	1.24 (1.13 to 1.37)		1.25 (1.14 to 1.37)		1.12 (1.01 to 1.24)		1.13 (0.96 to 1.33)		0.93 (0.69 to 1.26)	
≥1 hour/day	1.49 (1.25 to 1.77)		1.42 (1.19 to 1.69)		1.14 (0.94 to 1.39)		1.10 (0.82 to 1.48)		0.94 (0.63 to 1.39)	

^*^Statistical significance <0.05.

^†^Adjusted for age, sex, region of residence, the number of household, income level, educational level, physical activity [MPA], sleep time, stress level, and alcohol consumption, obesity, smoking status, and passive smoking.

A longer passive smoking duration at home (≥1 hour/day) was associated with wheezing (1.63 [1.39 to 1.91]; 1.49 [1.25 to 1.77]) and exercise wheezing (1.65 [1.42 to 1.93]; 1.42 [1.19 to 1.69]) in both workers and non-workers, respectively. A longer passive smoking duration (≥1 hour/day) in the workplace was also linked to wheezing (1.51 [1.39 to 1.65]) and exercise wheezing (1.59 [1.46 to 1.73]) in workers.

In the current and former smokers, as total pack-years of smoking increased (>30 pack-years), the incidences of wheezing (1.77 [1.61 to 1.93]), exercise wheezing (1.52 [1.39 to 1.66]) and current asthma (1.33 [1.15 to 1.53]) increased as well (Table 3). Total smoking time and the mean number of cigarettes per day showed similar results. An older age at smoking onset (≥26 years) was negatively correlated with wheezing (0.54 [0.48 to 0.61]), exercise wheezing (0.53 [0.47 to 0.60]), and current asthma (0.60 [0.49 to 0.74]). In the former smokers, the longer quitting duration of smoking (≥21 years) was inversely associated with wheezing (0.54 [0.48 to 0.61]), exercise wheezing (0.53 [0.47 to 0.60]), and current asthma (0.60 [0.49 to 0.74]).

**Table 3:** Adjusted odd ratios of each smoking pattern for asthma related questions in past and current smoker.

	Number (%^*^)	Wheezing		Exercise wheezing		Asthma ever		Asthma current	
		OR (95% CI) ^‡^	*P*-value	OR (95% CI) ^‡^	*P*-value	OR (95% CI) ^‡^	*P*-value	OR (95% CI) ^‡^	*P*-value
Cigarette			<0.001^†^		<0.001^†^		<0.001^†^		0.003^†^
1–10/day	121,219 (42.2)	1		1		1		1	
11–20/day	150,477 (47.6)	1.31 (1.23 to 1.40)		1.26 (1.19 to 1.34)		1.06 (0.99 to 1.14)		1.12 (1.01 to 1.25)	
21–30/day	19,885 (5.6)	1.68 (1.51 to 1.87)		1.55 (1.39 to 1.72)		1.25 (1.11 to 1.42)		1.28 (1.06 to 1.54)	
≥31/day	18,379 (4.6)	1.57 (1.40 to 1.75)		1.45 (1.30 to 1.62)		1.26 (1.12 to 1.43)		1.32 (1.12 to 1.57)	
Total smoking time			<0.001^†^		<0.001^†^		<0.001^†^		<0.001^†^
≤13 year	78,525 (32.7)	1		1		1		1	
14–22 year	77,736 (27.5)	0.95 (0.87 to 1.04)		0.91 (0.83 to 0.99)		0.67 (0.61 to 0.73)		0.92 (0.77 to 1.09)	
23–33 year	78,214 (23.8)	1.14 (1.04 to 1.25)		1.00 (0.91 to 1.09)		0.66 (0.60 to 0.73)		0.93 (0.79 to 1.10)	
≥34 year	7,548 (16.1)	1.85 (1.69 to 2.04)		1.65 (1.51 to 1.81)		1.03 (0.94 to 1.13)		1.30 (1.12 to 1.51)	
Total pack-year			<0.001^†^		<0.001^†^		<0.001^†^		<0.001^†^
≤7.25	77,575 (31.3)	1		1		1		1	
>7.25, ≤17	79,862 (28.0)	1.00 (0.91 to 1.09)		0.95 (0.87 to 1.04)		0.79 (0.72 to 0.87)		0.99 (0.84 to 1.16)	
>17, ≤30	80,116 (23.9)	1.28 (1.17 to 1.40)		1.18 (1.09 to 1.29)		0.85 (0.77 to 0.93)		1.18 (1.01 to 1.37)	
>30	72,407 (16.8)	1.77 (1.61 to 1.93)		1.52 (1.39 to 1.66)		1.08 (0.99 to 1.18)		1.33 (1.15 to 1.53)	
Quitting duration			<0.001^†^		<0.001^†^		0.005^†^		0.001^†^
1–5 year	28,009 (26.1)	1		1		1		1	
6–11 year	31,264 (28.2)	0.81 (0.71 to 0.92)		0.88 (0.78 to 1.00)		0.94 (0.82 to 1.07)		1.05 (0.88 to 1.26)	
12–20 year	29,244 (24.7)	0.70 (0.61 to 0.79)		0.73 (0.65 to 0.83)		0.86 (0.75 to 0.97)		0.87 (0.73 to 1.05)	
≥21 year	30,545 (21.0)	0.64 (0.56 to 0.73)		0.65 (0.58 to 0.74)		0.81 (0.71 to 0.92)		0.75 (0.63 to 0.90)	
Starting age			<0.001^†^		<0.001^†^		<0.001^†^		<0.001^†^
≤15 years-old	18,017 (6.1)	1		1		1		1	
16–19 years-old	106,807 (37.8)	0.75 (0.68 to 0.83)		0.76 (0.68 to 0.84)		0.78 (0.70 to 0.88)		0.86 (0.72 to 1.03)	
20–25 years-old	154,145 (48.4)	0.59 (0.54 to 0.65)		0.61 (0.55 to 0.68)		0.66 (0.59 to 0.74)		0.71 (0.60 to 0.85)	
≥26 years-old	30,991 (7.7)	0.54 (0.48 to 0.61)		0.53 (0.47 to 0.60)		0.59 (0.52 to 0.68)		0.60 (0.49 to 0.74)	

^*^Estimated prevalence adjusted recommended weighted value.

^†^Statistical significance <0.05.

^‡^Adjusted for age, sex, region of residence, the number of household, income level, educational level, physical activity [MPA], sleep time, stress level, and alcohol consumption, and obesity.

## Discussion

Both current and former smoking were positively related to wheezing, exercise wheezing, asthma ever, and current asthma in the present study. Wheezing and exercise wheezing were more closely associated with current smoking than former smoking. In contrast, a history of asthma and current asthma were more closely related to former smoking than current smoking. Passive smoking from the home and workplace also showed significant positive associations with wheezing and exercise wheezing. In addition, the cumulative and prolonged impact of smoking was noted according to the duration and amount of smoking. These results extend the findings of prior studies by demonstrating the association between smoking patterns, including the temporal and quantitative aspects and several asthma-related parameters.

Both current and former smoking were significantly associated with asthma-related parameters, including wheezing, exercise wheezing, asthma ever, and current asthma. The adverse effects of active smoking on lung function may contribute to asthmatic symptoms and asthma. In active smokers, the inhalation of tobacco smoke has been suggested to induce neutrophilic airway inflammation and airway remodeling by increased expression of several factors including placental growth factor, thereby accelerating airway obstruction and the decline of lung function in prospective clinical studies^14,15^. The present study presented the differential impacts of current or former smoking on asthma-related parameters. Current smoking was more closely associated with wheezing and exercise wheezing than former smoking. In contrast, former smoking was more closely related to asthma ever and current asthma than current smoking. It was presumed that the cumulative harmful effects of smoking on asthma could be better noted in former smokers. The unresolved harmful effects of smoking in former smokers were suggested in previous studies. The former smokers showed a persistently increased sputum microbiome diversity than that of current smokers^7^. The impaired lung function and eosinophilia were not relieved after 3 months of smoking cessation^8^. Additionally, it may take more time to diagnose asthma, although asthmatic symptoms were shown at early or subclinical stages of asthma. Furthermore, it is possible that a greater number of asthmatic patients quit smoking based on advice from health-care workers or by self-control to manage their airway disease due to the cross-sectional study design; however, this healthy smoker hypothesis is weakly supported because a previous study demonstrated that smokers with asthma did not show increased attempts to quit smoking by physician counseling or pharmacotherapy compared with healthy smokers^16^.

The aggravation of asthma was significantly related to active smoking status in the worker group only. Although the underlying mechanism is still elusive, it has been proposed that smoking increases occupational sensitization to many allergens, which probably provokes occupational asthma^17^. Several examples of occupational allergen sensitization in smokers have been reported, namely, laboratory animal allergens in laboratory workers, flour and amylase in bakers, and platinum salts in refinery workers^18^. The non-worker group may have been less exposed to stimuli that evoke asthmatic attacks than the workers. Undiagnosed asthma and chronic obstructive lung disease were also possible in the wheezing groups. In addition, the few subjects with aggravation of asthma or the non-workers may have influenced the statistical power.

Passive smoking was significantly associated with wheezing, exercise wheezing, and asthma ever in the present study. The side stream of tobacco smoke, which comprises the main portion of passive smoking, is harmful to upper airway function and aggravates asthma by several mechanisms such as modulating antioxidant functions^19^. Passive smoking causes poor responses to steroids in asthma patients by increasing the leukotriene level and oxidative stress-related mechanisms, such as histone deacetylase-2 function via activation of the phosphoinositide-3-kinase/Akt pathway^20,21^. In addition to the effects of side stream tobacco smoke, passive smokers are more likely to attempt active smoking^12^. Although we adjusted for current and former smoking to evaluate the independent effects of passive smoking on asthma, susceptibility to the active smoking experience could influence the adverse association between passive smoking and the asthma-related parameters. In the present study, passive smoking in the worker group was more significantly related to current asthma than that in the non-worker group. The attenuated association of passive smoking with current asthma may be a result of the smoke-free living environment of the asthmatic patients, as these patients may intentionally avoid passive smoking environments to benefit their health. In addition, the workers may have been more exposed to passive smoking than non-workers, despite their effort to avoid smoking.

The amount of smoking was significantly positively correlated with wheezing, exercise wheezing, asthma ever, and current asthma. The duration of smoking and total pack-years of smoking were positively associated with wheezing, exercise wheezing, and current asthma. A previous study also demonstrated the proportionally decreased lung function in subjects with a greater number of pack-years of smoking^22^. In addition, the present results also showed that a longer duration of smoking cessation was inversely related to wheezing, exercise wheezing, asthma ever, and current asthma. Thus, both the amount and duration of smoking are likely to play a role in asthma manifestations. The age at smoking onset also affected the relationship between smoking and asthma. The younger the age at smoking onset, the more patients experienced wheezing, exercise wheezing, asthma ever, and current asthma in the present study. The vulnerability of the developing lung to harmful stimuli may be attributed to the increased impact of smoking on asthma in those who start smoking early. Indeed, the maturation of lung, immune, and repair function in youth may be affected by smoking^23^. In addition, the longer duration and amount of smoking in early smokers may influence these relationships, although we adjusted for the age of the participants.

The present results should be interpreted with consideration of some weak points. The cross-sectional study design limited the causality between smoking and asthma. The survey was based on self-reported questionnaires, which are subject to recall bias. We could not conduct objective lung function tests. The information on previous and current asthma was based on the history of diagnosis by a clinician. Thus, it was possible that there were misdiagnosed or under-diagnosed patients in our cohort; however, the survey on asthma was standardized by the use of International Study of Asthma and Allergies in Childhood questionnaires.

Several aspects of the present study render it superior to previous studies. Most importantly, this study is one of the largest, representative population-based studies on smoking and asthma. The validity of the data was ensured by the repeated consistency with several previous studies. Age, region of residence, type of housing, income level, education level, physical activity, obesity, sleep time, stress level, and alcohol intake were adjusted for to minimize possible confounding effects. Moreover, we conducted additional subgroup analyses in accordance with obesity (Supplementary Tables 1–3). Smoking can influence asthma mediated by obesity because obesity is associated with both smoking and asthma (Supplementary Tables 1 and 2); however, all the subgroups (underweight, healthy, overweight, and obese) showed significant positive relationships between smoking and asthma (Supplementary Table 3). Importantly, various smoking statuses, including current and former smoking, as well as duration, amount, and onset age of smoking, were considered. Asthma-related symptoms were also divided into categories, such as wheezing, exercise wheezing, recent aggravation, current asthma and history of asthma. These classifications assisted in specifying the associations between smoking and asthma symptoms, which were consistent irrespective of obesity.

## Methods

### Study population and data collection

This study was approved by the Institutional Review Board of Korea Centers for Disease Control and Prevention (KCDC) (IRB No. 2010-02CON-22-P, 2011-05CON-04-C, and 2013-06EXP-01-3C). Written informed consents were obtained from all the participants prior to the survey. All Korean Community Health Survey (KCHS) data analyses were conducted in accordance with the guidelines and regulations provided by the KCDC.

This study is the cross-sectional study using the data from the KCHS. This study covers one nation using statistical methods based on designed sampling and adjusted weighted value. The KCHS conducted in 2009, 2010, 2011, and 2013 were analyzed. The survey gathers information through face-to-face, paper assisted personal interviews between trained interviewers and respondents. The sample size for the KCHS is 900 subjects in each of 253 community units, including 16 metropolitan cities and provinces. The KCHS uses a two-stage sampling process clustered sampling. The first stage selects a sample area (tong/ban/ri) as a primary sample unit, which is selected according to the number of households in the area using a probability proportional to the sampling method. In the second stage, the number of households in the selected sample tong/ban/ri is identified to create a household directory. Sample households are selected using systematic sampling methods. This process is used to ensure that the sample units are representative of the entire population^24^. For the sample to be statistically representative of the population, the data collected from the survey were weighted by statisticians who performed post-stratification and considered the non-response rates (Supplementary note 1)^25^.

**Figure 1:**
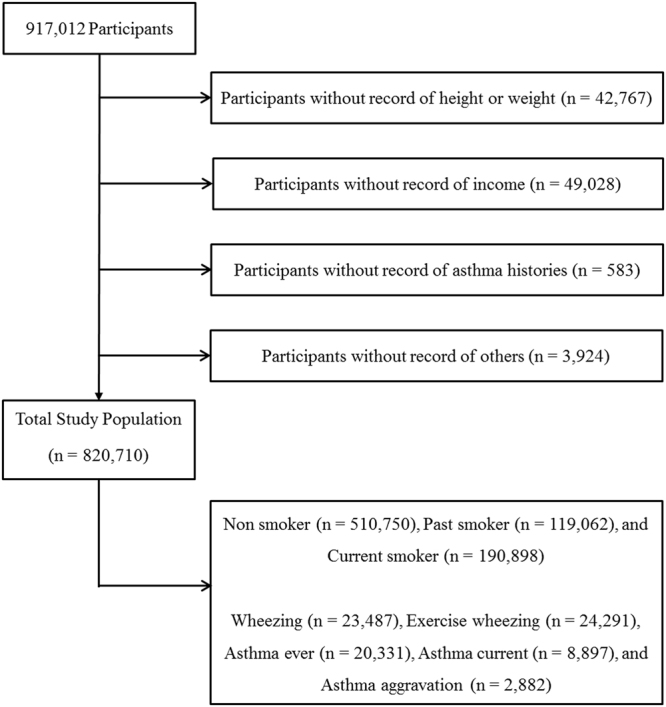
A schematic illustration of participant selection in the present study.

Of the 917,012 total participants, we excluded the following participants in this study: the participants with a height less than 110 cm or weight less than 30 kg or who did not report their height or weight (43,383 participants); the participants who did not report their income (49,028 participants); the participants who did not report answers to the smoking questions (2,588 participants); the participants who did not report answers to the asthma-related questions (583 participants); and the participants who did not report answers to other survey questions (765 participants) (Fig. 1). A total of 820710 participants, with ages ranging from 19 to 109 years, were included in this study. Among them, 551,801 participants were grouped as workers and 268,909 were grouped as non-workers (i.e., full-time housewives, students, and unemployed individuals) according to their occupation histories.

## Questionnaire

### Independent variables

The participants were divided into 3 groups according to smoking status: non-smokers, former smokers, and current smokers. The non-smokers were defined as a participant who has never smoked, or who has smoked less than 100 cigarettes in his or her life time. the former smokers were defined as a participant who has smoked at least 100 cigarettes in his or her lifetime and quit smoking ≥1 year prior to this study. The former smokers who quit smoking less than 1 year prior to the study were included in the current smokers group. Smoking patterns, the number of cigarettes per day and the number of smoking days per month were assessed. The individuals were divided into 4 groups according to the mean number of cigarettes per day as follows: 1–10, 11–20, 21–30, and ≥31 cigarettes per day. Total smoking time (years) was divided into four quartiles: ≤13 years; 14–22 years; 23–33 years; and ≥34 years. Total pack-years were calculated by multiplying the number of packs smoked per day by the total smoking time and divided into four quartiles: ≤7.25; >7.25, ≤17; >17, ≤30; and >30. The age at smoking onset was categorized into four groups: ≤15 years, 16–19 years, 20–25 years, and ≥26 years. For the former smokers, the periods of smoking cessation were divided into 4 quartiles: 1–5 years, 6–11 years, 12–20 years, and ≥21 years. Passive smoking in the home and workplace was categorized into 3 groups based on the time of exposure to cigarette smoke from another person: 0 hours per day, <1 hour per day, and ≥1 hour per day. Passive smoking in the workplace was not surveyed among non-workers.

### Dependent variables

The participants were asked asthma-related questions, such as “Have you had wheezing or whistling in the chest in the past 12 months?”, “In the past 12 months, has your chest sounded wheezy during or after exercise?”, “Have you ever been diagnosed with asthma by a doctor?”, “In the past 12 months, have you been treated for asthma by a doctor?”, and “In the past 12 months, have you visited the emergency room or the hospital abruptly because of asthma symptoms?” Participant who has ever been diagnosed with asthma by a medical doctor were recorded as asthma ever as a previous study^26^. The current asthma was defined as a participant who has been treated for asthma by a doctor less than 1 year prior to the study as a previous study^26^.

### Covariates

The region of residence was divided into 2 groups according to administrative district: urban (i.e., Seoul, Gyeonggi, Busan, Daegu, Incheon, Gwangju, Daejeon, Ulsan, and Sejong) and rural areas (i.e., Gangwon, Chungbuk, Chungnam, Jeonbuk, Jeonnam, Gyeongbuk, Gyeongnam, and Jeju). The number of occupants in the household was surveyed. The type of residence was divided into 3 groups: a detached house, condominium, and others. Using the methods recommended by the Organization for Economic Cooperation and Development^27^, (i.e., dividing household income by the square root of the number of household members) monthly income was divided into the lowest, low-middle, upper-middle, and highest quartiles. To explore the influence of educational level, uneducated participants and those who had graduated only from elementary or middle schools were assigned to the “low” education group; high school-educated individuals comprised the “middle” education group; and junior college, college and graduate school graduates constituted the “high” education group.

To assess physical activity intensity, the participants were asked “How often do you perform light or moderate leisure time physical activities for at least 10 min that cause only light sweating or a slight to moderate increase in breathing or heart rate?” The participants reported on both the frequency and duration of moderate physical activities. To assess the amount of vigorous-intensity physical activity performed, the participants were asked “How often do you perform vigorous leisure time physical activities for at least 10 min that cause heavy sweating or large increases in breathing or heat rate?” The participants reported on both the frequency and duration of vigorous physical activities. One minute of vigorous activity was counted as two minutes of moderate activity. The sum of vigorous and moderate physical activities was measured as moderate-intensity physical activity (MPA). The MPA category was divided into 3 groups: 0 minutes/week (inactive); 1–149 minutes/week (insufficiently active); ≥150 minutes/week (sufficiently active)^28^.

Using the international classification of adult underweight, overweight and obesity according to body mass index (BMI, kg/m^2^) of World Health Organization^29^, four BMI groups were devised: underweight, <18.5; healthy, ≥18.5, <25; overweight ≥25, <30; obese, ≥30. Amount of sleep was divided into 5 groups: 3–5 hour per day, 6 hour per day, 7 hour per day, 8 hour per day, and ≥9 hour per day. Participants who sleep less than 3 hours were excluded in this study. The participants were asked whether they usually feel stress, and the stress level was divided into the following four groups: no stress, some stress, moderate stress, and severe stress. Alcohol consumption was divided into the following three categories: ≤1 time a month; 2–4 times a month; ≥ 2 times a week.

### Statistical analysis

The estimated prevalence is presented as weighted values according to the recommendations of KCHS.

Adjusted odd ratios (AORs) of smoking status and passive smoking in workers and non-workers were calculated for the asthma-related questions using multiple logistic regression adjusted for the independent variables.

The AORs of smoking for obesity were calculated using polychotomous logistic regression adjusted for the independent variable groups. The AORs of obesity for the asthma-related questions were calculated using multiple logistic regression adjusted for the independent variable groups. For the subgroup analysis according to obesity, the AORs of smoking for the asthma-related questions were calculated using multiple logistic regression adjusted for the independent variable groups.

The AORs of each smoking pattern in former and current smokers were calculated for the asthma-related questions using multiple logistic regression adjusted for the independent variable groups and obesity.

Two-tailed analyses were conducted and *P*-values lower than 0.05 were considered to indicate significance. 95% confidence interval (CI) was calculated. All AORs were calculated with taking into account the complex sampling structure of the data. The missing participants were calculated as the valid missing participants to use weighted values. The results were analyzed statistically using SPSS ver. 21.0 (IBM, Armonk, NY, USA).

### Data availability

The data underlying this study cannot be made public. All of data are available from the database of Korea Community Health Survey (KCHS) (http://KCHS.cdc.go.kr/). KCHS controls access to this data and grants access to any researcher who follows established ethical approval process outlined by KCHS.

## Electronic supplementary material

Supplementary note
