# Supplementary material for: Active and passive smoking impacts on asthma with quantitative and temporal relations: A Korean Community Health Survey
Source: Sci Rep. 2018 Jun 5;8:8614. doi: 10.1038/s41598-018-26895-3 (PMC5988685; doi:10.1038/s41598-018-26895-3)
Supplement: Supplementary file 1 — Supplementary note [file 41598_2018_26895_MOESM1_ESM.pdf]

**Active and passive smoking impacts on asthma with quantitative and temporal relations: A Korean Community Health Survey**

So Young Kim, MD[1], Songyong Sim, PhD[2], Hyo Geun Choi, MD[3*]

[1]Department of Otorhinolaryngology-Head & Neck Surgery, CHA Bundang Medical Center, CHA University, Seongnam, Korea

[2]Department of Statistics, Hallym University, Chuncheon, Korea

[3]Department of Otorhinolaryngology-Head & Neck Surgery, Hallym University Sacred Heart Hospital, Anyang, Korea

**Corresponding.** pupen@naver.com

**Supplementary note 1 Description**

In this study, the participants were weighted using this method to represent mother populations. Weighted values were calculated using household weight, personal weight, and adjusted weight.

(1) Household weight were calculated as below

$W_{hi} = M_{hi}/n_{hi} = e_{hi} * N_{hi} /n_{hi}$

$N_{hi}$ = i type household number in h (tong/ban/ri) descrict

$M_{hi}$ = i type appropriate household number in h (tong/ban/ri) descrict

$n_{hi}$ = i type survey household number in in h (tong/ban/ri) descrict

$e_{hi}$ = i type appropriate survey household number in h (tong/ban/ri) descrict

(2) Personal weight were calculated as below

$W_{hjk} = W_{hjk} * P_{hjk}/p_{hjk}$

h: (tong/ban/ri) descrict

j: smple are

k; household

l; the number of household member

$P_{hjk}$: the number of household ≥ 19 years old

$p_{hjk}$: the number of survey household ≥ 19 years old

(3) Adjusted weight were calculated as below to adjust population structure according to the resident registration number

$W`_{hijkl} = BF_{d} * W_{hijkl}$

$BF_d$ = d (the number of population according to the resident registration number according to age and sex)/($\sum_h \sum_i \sum_{jkl} W_{hijkl} I_d \in_{\text{according to age, sex}}$)

The fidelity of the data was annually verified and reported as the Quality Control and Evaluation for Community Health Survey.

**Supplementary Table S1 Subgroup analyses of adjusted odd ratios of smoking status for asthma related questions according to obesity**

| | Wheezing | | Exercise wheezing | | Asthma ever | | Asthma current | |
|---|---|---|---|---|---|---|---|---|
| | OR (95% CI) † | *P*-value | OR (95% CI) † | *P*-value | OR (95% CI) † | *P*-value | OR (95% CI) † | *P*-value |
| **Underweight** | | | | | | | | |
| Active smoking status | | <.001* | | <.001* | | <.001* | | <.001* |
| Non-smoker | 1 | | 1 | | 1 | | 1 | |
| Past smoker | 2.10 (1.64 to 2.68) | | 1.93 (1.52 to 2.44) | | 2.25 (1.75 to 2.89) | | 2.41 (1.69 to 3.43) | |
| Current smoker | 2.06 (1.67 to 2.53) | | 1.88 (1.49 to 2.38) | | 1.90 (1.48 to 2.43) | | 1.76 (1.25 to 2.49) | |
| **Healthy** | | | | | | | | |
| Smoking status | | <.001* | | <.001* | | <.001* | | <.001* |
| Non-smoker | 1 | | 1 | | 1 | | 1 | |
| Past smoker | 1.66 (1.52 to 1.82) | | 1.64 (1.50 to 1.79) | | 1.62 (1.48 to 1.78) | | 2.15 (1.87 to 2.48) | |
| Current smoker | 2.34 (2.15 to 2.54) | | 1.92 (1.77 to 2.09) | | 1.28 (1.16 to 1.41) | | 1.72 (1.47 to 2.00) | |
| **Overweight** | | | | | | | | |
| Smoking status | | <.001* | | <.001* | | <.001* | | <.001* |
| Non-smoker | 1 | | 1 | | 1 | | 1 | |
| Past smoker | 1.55 (1.35 to 1.78) | | 1.49 (1.30 to 1.71) | | 1.58 (1.36 to 1.83) | | 1.72 (1.35 to 2.19) | |
| Current smoker | 2.56 (2.26 to 2.90) | | 2.08 (1.83 to 2.36) | | 1.44 (1.24 to 1.66) | | 1.60 (1.24 to 2.06) | |
| **Obese** | | | | | | | | |
| Smoking status | | <.001* | | <.001* | | .173 | | .009* |
| Non-smoker | 1 | | 1 | | 1 | | 1 | |
| Past smoker | 1.14 (0.82 to 1.60) | | 1.28 (0.94 to 1.75) | | 1.34 (0.95 to 1.89) | | 2.35 (1.36 to 4.06) | |
| Current smoker | 1.86 (1.36 to 2.52) | | 1.74 (1.32 to 2.31) | | 1.04 (0.73 to 1.47) | | 1.58 (0.90 to 2.78) | |

* Statistical significance <.05

† Adjusted for age, sex, region of residence, the number of household, income level, educational level, physical activity [MPA], sleep time, stress level, and alcohol consumption, smoking status, and passive smoking.

**Supplementary Table S2 Odd ratios of smoking for obesity (Reference = Non-smoker)**

| | OR (95% CI) | *P*-value |
|---|---|---|
| | | <.001* |
| Past smoker | | |
| Underweight | 1.38 (1.30 to 1.46) | |
| Healthy | 1 | |
| Overweight | 1.17 (1.14 to 1.20) | |
| Obese | 1.16 (1.08 to 1.26) | |
| Current smoker | | |
| Underweight | 1.38 (1.31 to 1.45) | |
| Healthy | 1 | |
| Overweight | 1.04 (1.02 to 1.07) | |
| Obese | 1.18 (1.11 to 1.26) | |

* Statistical significance < .05

**Supplementary Table S3 Odd ratios of obesity for asthma**

| Obesity | Wheezing | | Exercise wheezing | | Asthma ever | | Asthma current | |
|---|---|---|---|---|---|---|---|---|
| | OR (95% CI) | *P*-value | OR (95% CI) | *P*-value | OR (95% CI) | *P*-value | OR (95% CI) | *P*-value |
| | | <.001* | | <.001* | | <.001* | | <.001* |
| Underweight | 1.46 (1.35 to 1.56) | | 1.43 (1.33 to 1.53) | | 1.15 (1.06 to 1.24) | | 1.44 (1.29 to 1.60) | |
| Healthy | 1 | | 1 | | 1 | | 1 | |
| Overweight | 1.36 (1.31 to 1.42) | | 1.30 (1.25 to 1.36) | | 1.21 (1.16 to 1.27) | | 1.19 (1.11 to 1.28) | |
| Obese | 2.28 (2.07 to 2.51) | | 2.42 (2.21 to 2.66) | | 2.04 (1.83 to 2.27) | | 2.12 (1.78 to 2.52) | |

* Statistical significance < .05
